# Wrecked Clergymen

**Published:** 1861-05

**Authors:** 


**WRECKED CLERGYMEN.**—The utter stupidity of the men who have the control and management of Theological Seminaries, is inconceivable, and the young men who go there do, in the judgment of charity, possess more piety than brains; their zeal is not according to knowledge; they cheat themselves, they cheat their parents, and they cheat the church. A considerable part of the money which is expended in paying professors' salaries and in sustaining "poor and pious young men," comes from people who have to work for a living; from farmers, mechanics; from girls and old women who make it by sewing and knitting; from persons who, by severe economies, lay up a little as a matter of duty and of love to the Master. As faithful stewards, the men who distribute this money, or who order its distribution, are bound to do it wisely, to the best advantage. If this is done by throwing out a bran-new preacher on the community who has barely strength to stand up long enough to read a sermon, who is so wrecked in brain and ruined in body, that it is problematical whether either will last six months from the day of his leaving the seminary—then we do not know what a faithful stewardship is. A professor in a theological seminary has no sense, never had any, never will have, beyond what he gathers in chewing Hebrew roots and ferretting out Greek themes; his whole soul is taken up in proving doctrines that never existed except in his own imagination; in showing what can not be seen; in fabrications which a breath of truth will smash to atoms. And looking forward to the time when they shall be quoted as very giants in Scriptural learning, they seem wholly to forget that the young men under their care have something else to do besides cramming their brains with theological abstractions. It really admits of debate, whether it would not be better for the world if the engine of Time had not better take a turn back by dumping every theological seminary, every medical college, and every public school into Symmes' north hole, and make another start in the direction of a better progress. Meanwhile, let this young man who wants an education, work by day for his board and tuition, and study at night by the light of a pine-knot or a bit of a rag dipped in a saucer of hog's fat. We have used both, and know their virtues. Let the other youngster who wants to become a doctor, take up the old-fashioned plan of compounding his master's medicines, going round with him to visit patients, and gradually learn to take his place when emergencies present themselves, thus making actual observation the foundation of his study and his skill. And as to theological training, certain it is that some of our greatest divines, the men who have signalized themselves by splendid utilities, were those who plodded while they plowed, who "studied" with their own ministers, and thus literally graduated from "the school of the prophets," without being hurried to death to keep up in the class with those who had brains and nothing else. The *Pittsburgh Missionary* gives a telling illustration of one of the points made above as follows, and we could make a formidable list akin to it, from our own personal observation.

"Scarcely two years ago, I was summoned to the grave of my brother, Rev. ——, who died with consumption in the very prime of manhood, with the most gratifying evidences of usefulness behind him, and the most encouraging prospects of continued usefulness before him. Soon after the failure of his health, I was attacked almost in the same way. A visit to the South had some influence in restoring my health, yet I can not now actively engage in the ministry. This, I assure you, afflicts me more than the pain arising from the disease itself. Whilst I hope I am gradually improving, another brother younger than myself, who has been studying for the ministry, is now passing through the last stage of consumption! In his great eagerness to acquire an education in a short time, he broke down his health. I will soon be the last of the family. My father died twenty-five years ago, and his last request was, that his three sons should be trained for the ministry. My excellent mother, true to her promise, made every sacrifice possible for a mother, to carry out his wish. My brothers went to the University of Virginia to acquire an education. They acquitted themselves with honor, but returned home with broken constitutions! My censure may be unfair, but it seems to me that the learned Faculty of that Institution are blest with but a small degree of knowledge in regard to the laws of health, or have not philanthropy enough to render practical what they do know concerning the laws of the body. I know many young men who have graduated there with distinction, who are now among the dead. Half-fed, half-warmed, pushed, pulled, drilled, and drummed to learn rapidly—no attention paid to regular exercise, and, in consequence, the health of the poor student sinks. Surely, such learned men ought to know that exercise is indispensable, and that if students are too indifferent or parsimonious of time to take it, they should be compelled to do so, just as the student is compelled to recite his lesson at a certain hour. This system of "all work and no play," has filled my mind with sad thoughts, in regard to the future of many of our young ministers."

